# Pupil Reactions to Tactile Stimulation: A Systematic Review

**DOI:** 10.3389/fnins.2021.610841

**Published:** 2021-02-22

**Authors:** Mariana de Mello Gusso, Gabriele Serur, Percy Nohama

**Affiliations:** Laboratório de Engenharia de Reabilitação, Programa de Pós-Graduação em Tecnologia em Saúde, Escola Politécnica, Pontifícia Universidade Católica Do Paraná, Curitiba, Brazil

**Keywords:** pupil dilation, tactile stimulation, perception, eye-tracking, norepinephrine system

## Abstract

Pupil dynamics can represent an indirect measure of perception; thus, it has been broadly explored in the auditory and visual fields. Although it is crucial for experiencing the outside world, tactile perception is not well-explored. Considering that, we sought to answer the following question via a systematic review: does normal tactile perception processing modulate pupil dilation in mammals (human or not)? The review process was conducted according to PRISMA Statement. We searched on Periódicos CAPES (Brazil) for the following terms: [(touch) OR (cutaneous stimulation) OR (tactile perception) OR (somatosensory) AND (pupil OR pupillary) NOT blind NOT reflex NOT pain NOT fear NOT noxious NOT autism NOT nerve NOT (pupillary block) NOT glaucoma NOT cataract NOT aneurysm NOT syndrome NOT treatment NOT special education]. From the 6,488 papers found, 4,568 were duplicates, and nine fulfilled the inclusion criteria. All papers found a positive relationship between pupil diameter and tactile perception. We found that the pupil is a reliable indirect measure of brain states and can evaluate norepinephrine (NE)/locus coeruleus (LC) action, stimulus inhibition, arousal, cognitive processes, and affection independently of the stimuli category (visual, auditory, or tactile). We also found that the perceptual tactile processing occurs in similar ways as the other perceptual modalities. We verified that more studies should be done, mostly avoiding low sampling rate recording systems, confounders as cue signs, not automated stimulation, and concurrent stimulus and using more reliable equipment.

## Introduction

To understand the neural basis of consciousness—one of the main goals of modern science (Miller, 2005; Michel et al., 2019)—it is necessary to approach perception in its different modalities. The visual (mainly) and auditory domains have been broadly investigated (Del Cul et al., 2007; Wyart and Tallon-Baudry, 2008; Gaillard et al., 2009; Carbon and Jakesch, 2012; Li et al., 2014; Pitts et al., 2014; Carbon, 2016; Herman et al., 2019). However, there is a lack of study on tactile perception. Proof of this disparity is when comparing searches on the three fields. The search on Periódicos CAPES (a search engine that searches on several databases like Web of Science, PubMed, Elsevier, and OneFile), from the 163,356 papers found on “conscious perception,” 35.24% are on visual, 10.85% on auditory, and only 7.56% on tactile/somatosensory conscious perception. Carbon (2016) and Carbon and Jakesch (2012) state that visual dimension dominates the comprehension of the perceptual phenomena and they urge the scientific field to pay more attention to tactile/haptic perception and broaden the understanding of perception.

Some of the methods of studying the nervous system are functional magnetic resonance imaging (fMRI), scalp electroencephalography (sEEG), magnetoencephalography (MEG), intracranial EEG (icEEG), and pupillometry. fMRI can produce whole-brain images showing contrasts. However, it does not have a high temporal resolution. sEEG and MEG have a higher temporal resolution but are affected by external artifacts and have a low spatial resolution, not accessing deep structures. The method with both good spatial and temporal resolution is icEEG since it records directly from the brain, but it is hard to conduct with humans since it must be done during necessary surgical electrode implant (Herman et al., 2019). Pupillometry is a non-invasive and complementary measure of cognition with a high temporal resolution (Luna et al., 2008; Tatler et al., 2014; Eckstein et al., 2017) that can be an indicator of perception and so a tool for developing no-report paradigms that should avoid the response confounder (Einhauser et al., 2010; Piquado et al., 2010; Laeng and Endestad, 2012; Laeng et al., 2012; Kang and Wheatley, 2015).

Eye metrics are the measurement and evaluation of eye and eyelid dynamics. They provide an ideal and powerful objective measure of ongoing cognitive processes and information requirements during behavior. They are non-invasive, have a high temporal resolution, and are a well-understood neural foundation, providing an ideal neuroscience model to investigate the association between brain mechanisms and behavior (Luna et al., 2008; Tatler et al., 2014; Eckstein et al., 2017). The process of pupil measurement that was once time-consuming is relatively easy to carry out and non-invasive today, having a spatial resolution (in diameter) better than 0.025 mm on individual measurements at sampling rates of 25–2,000 Hz (Granholm and Steinhauer, 2004; Eckstein et al., 2017) or lower when using webcam-based systems (Holmqvist et al., 2011; Schriver et al., 2018, 2020).

Pupillometry measures variations in the diameter of the pupillary aperture of the eye in response to physiological or psychological stimuli (Granholm and Steinhauer, 2004; Laeng et al., 2012). Pupil size is changed by two antagonistic muscles: the dilator pupillae and the sphincter pupillae. The sphincter muscle receives input from brain systems involved in pupillary light reflex, and both muscles receive inputs from brain systems involved in cognitive and autonomic functions, being influenced by it (Bremner and Spence, 2017; Eckstein et al., 2017).

Pupil dilation is directly related to conditions of increased attention or cognitive load or of emotional or cognitive arousal. Pupillometry is proven to work as an indicator of perception, language processing, memory and decision making, emotion and cognition, and cognitive development (Sirois and Brisson, 2014).

One explanation for the link between pupil dilation and psychological and physiological stimuli is that the dilation can be attributed to the sympathetic system's activation during autonomic arousal and mental activity, being modulated by the noradrenergic locus coeruleus (LC) (Aminihajibashi et al., 2020). The LC is essential for the regulation of physiological arousal and cognitive functioning. It produces the neurotransmitter norepinephrine (NE) and has direct inhibitory projections to the parasympathetic Edinger–Westphal nucleus, where the pupil's constricting fibers originate, therefore also inhibiting its constriction and indirectly enabling the pupil's dilation. LC also stimulates the sympathetic system, including the fibers that innervate the pupil to dilate it (Sirois and Brisson, 2014; Eckstein et al., 2017).

Pupil study is crucial since it may benefit the evaluation of “special populations who may not be able or willing to provide a typical behavioral answer (complex motor or verbal responses) to certain research questions, such as pre-verbal infants, non-verbal adults, or children with ASD” (autistic spectrum disorder) (Sirois and Brisson, 2014). The close relationship between task-evoked pupil dilation and its underlying neural mechanisms enable the use of this method with participants of any age; knowledge about this relationship allows researchers to relate the neural system and cognitive studies and to interpret results of cognitive studies in terms of underlying neurophysiological processes (Eckstein et al., 2017; Medathati et al., 2020).

Visual and auditory stimuli may cause pupil dilation response both in adults and in infants (Aston-Jones and Cohen, 2005; Einhauser et al., 2010; Piquado et al., 2010; Laeng and Endestad, 2012; Kang and Wheatley, 2015; Wetzel et al., 2016; Eckstein et al., 2017). Moreover, it appears to be affected by arousal rather than attention itself since its reaction to a cue was not a predictor of better performance on a visual task (Aminihajibashi et al., 2020). Pupillary light reflex can be artificially created by showing the sun's pictures, revealing the top-down effects of perception in the pupil dilation. Its properties, like delay, speed, and length of a change in pupil diameter index, affect various aspects of attention and memory (Sirois and Brisson, 2014).

Tactile perception, popularly known as “touch,” is the first of our senses to develop (Bremner and Spence, 2017; Miguel et al., 2019). Through the sense of touch, one perceives their own body and develops a sense of self (Carbon and Jakesch, 2012, Bremner and Spence, 2017; Hoffmann et al., 2017; Rigato et al., 2019). Moreover, touch is essential for developing social function (Schneider et al., 2016; Bremner and Spence, 2017; Cascio et al., 2019).

Tactile perception differs from haptic exploration since it does not include movement. The haptic exploration [which encompasses tactile and kinesthetic sense (Lederman and Klatzky, 2009)] gives us access to physical objects and the external world (Hoffmann et al., 2017). Carbon and Jakesch (2012) developed the haptic aesthetic processing model. They hypothesize that the haptic aesthetic experience occurs in three levels. The first is a low level of exploration, the second is a mid-level of assessment, and the last is a high level of evaluation, where the cognitive and emotional processing occurs. There is a feedback loop in each of these levels (expectation, integration, and familiarity, respectively). There are different pupil reactions to these different levels, but further studies need to be done to correlate them to that model.

We rarely use passive tactile perception alone in daily life since we explore the environment to get to know it. Differently from other sensory modalities, the touch is an active process, since what is touched touches back the person who is touching it (Carbon and Jakesch, 2012). In research, if we want to understand the neural correlations of perception, we need to isolate it, study touch (lower level processing) and movement separately, avoiding signal noise between the two, and, later, integrate both in the study of haptics. Because of tactile perception's relevance for social and cognitive domains, it should be placed more centrally in the study of perception than it is.

Tactile stimuli can be delivered manually (Van Hooijdonk et al., 2019) or in an automated manner. The most common delivery materials are brushes for affective touch (Loken et al., 2011; Croy et al., 2014; Sailer et al., 2016; Hielscher and Mahar, 2017; Van Hooijdonk et al., 2019), electrical stimulation (Mückschel et al., 2020), or mechanical stimulation that can be delivered by different actuators (Garcia-Hernandez et al., 2014): piezoelectric (Schriver et al., 2018, 2020; Ganea et al., 2020; Lee et al., 2020), pneumatic (Moy et al., 2000; Yoo et al., 2015), electrical motor (Sarakoglou et al., 2012, 2014), ultrasonic (Cugini et al., 2009; Kim et al., 2009; Bordegoni et al., 2010), shape memory alloy (SMA) (Velázquez et al., 2006, 2008; Biet et al., 2008), and micro-actuators based on micro-electro-mechanical systems (MEMS) technologies (Ninomiya et al., 2009, 2011; Sarakoglou et al., 2012, 2014; Streque et al., 2012).

Although pupillometry can be such a powerful tool to indirectly measure cognitive processes and brain activity, the studies relating it to tactile perception are still scarce but relevant. It is essential to know if the findings in other perceptual fields can be translated to tactile perception searching for an integrative perception theory. This way, we present the existing findings that look for clarifying pupil interaction dynamics and tactile stimulation.

## Methods

Based on the PICOS strategy (Liberati et al., 2009, Figure 1), we sought to answer whether normal tactile perception modulates pupil dilation in mammals (human or not); the review was conducted according to the PRISMA Statement (Liberati et al., 2009; Moher et al., 2009, Figure 2).

**Figure 1:**
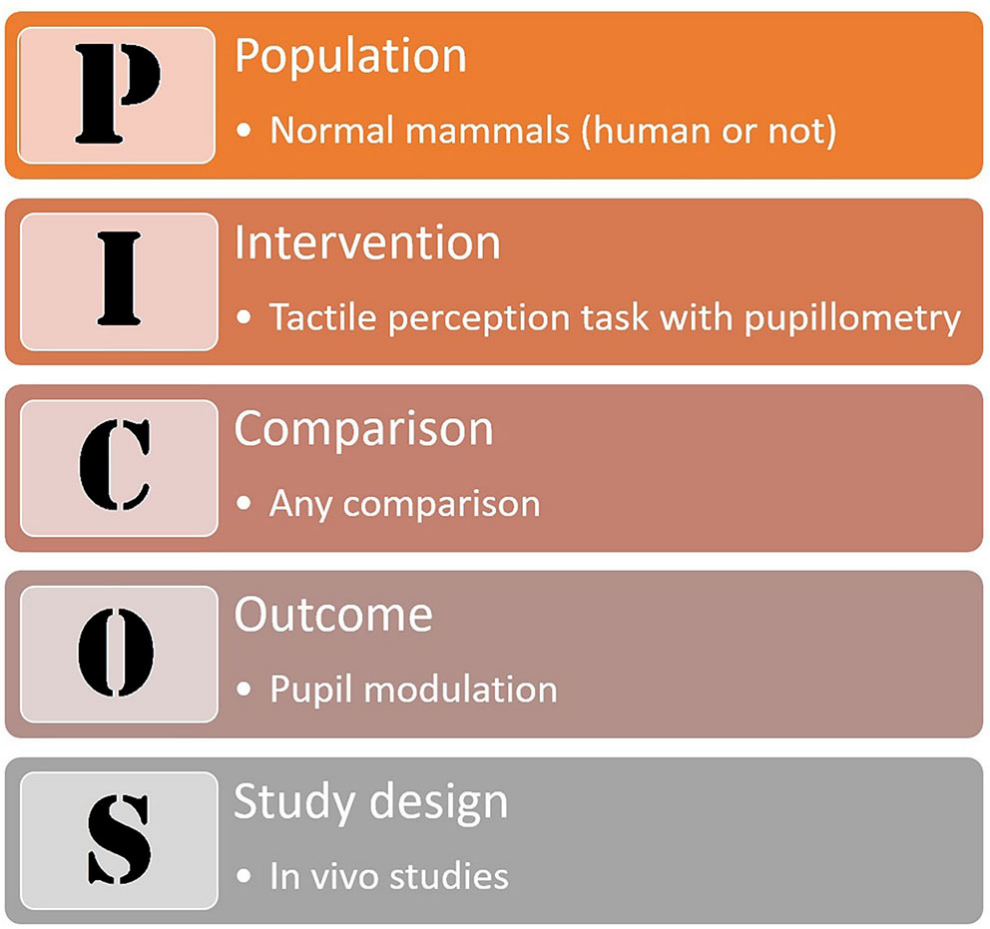
The PICOS strategy.

**Figure 2:**
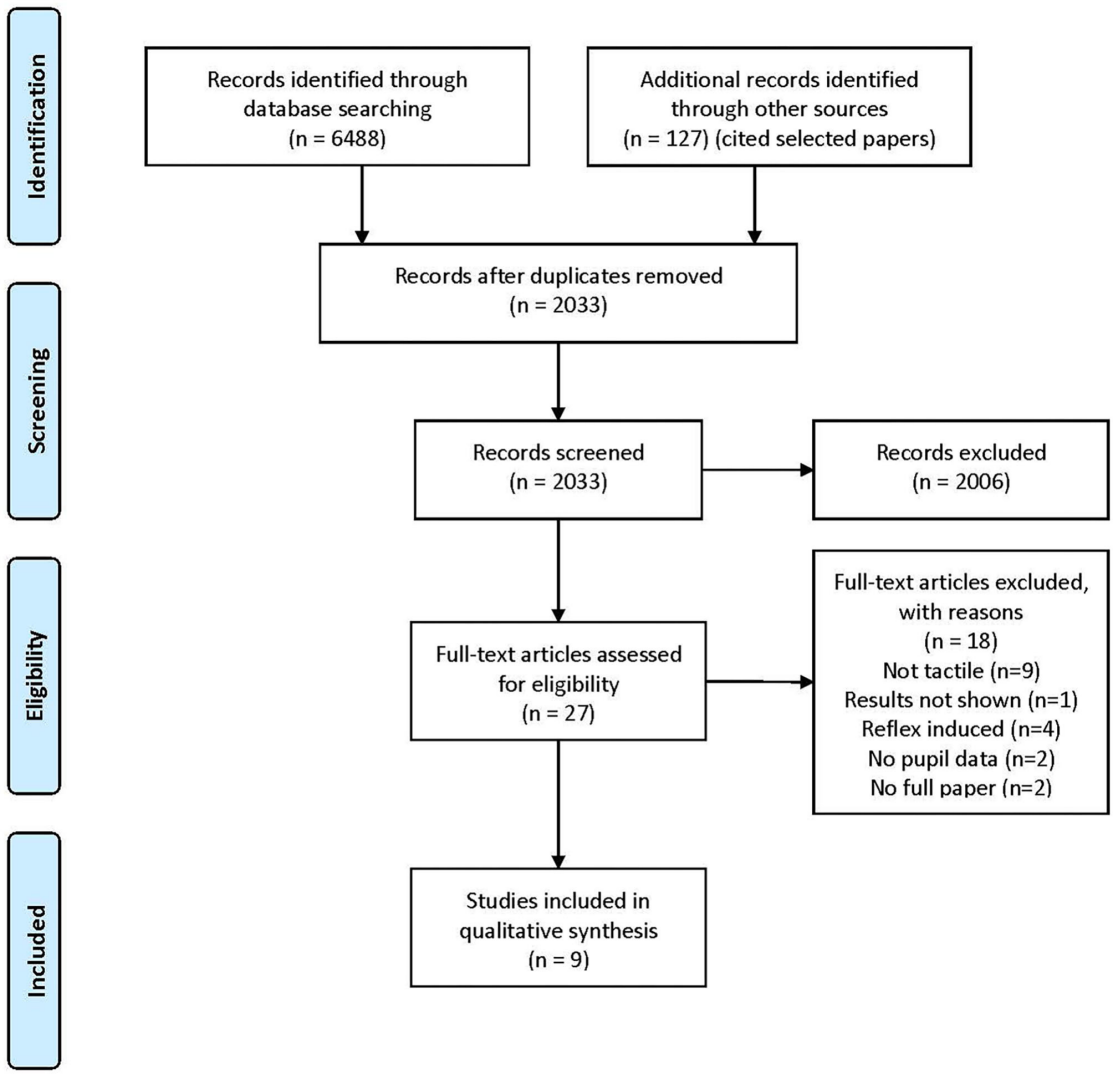
PRISMA 2009 flow diagram. The figure shows the PRISMA 2009 flow diagram with the steps for selecting the papers considered for review: identification, screening, eligibility, and inclusion. The additional records identified through other sources are the ones found by Google Scholar that cited the reviewed studies.

To find the papers that would help us answer this question, we used Periódicos CAPES' (https://www.periodicos.capes.gov.br/) search engine. This engine searches several databases at the same time. We used the term {[touch OR (cutaneous stimulation) OR (tactile perception) OR somatosensory] AND (pupil OR pupillary) NOT blind NOT reflex NOT pain NOT fear NOT noxious NOT autism NOT nerve NOT (pupillary block) NOT glaucoma NOT cataract NOT aneurysm NOT syndrome NOT treatment NOT special education} for this search. We restricted the search to only peer-reviewed full-length papers published until November 2020. After selecting the papers that fitted the selection criteria, we used Google Scholar to find the ones that cited them and included those in the research.

### Eligibility Criteria

From the files found, we selected the ones that were full-length peer-reviewed papers and were conducted with mammals (human or not) with no medical condition. We excluded the ones that were about noxious stimulation, toxicology, or normal or pathological reflex.

The studies' selection was conducted by two researchers independently that analyzed titles and abstracts and selected papers for full-text appreciation.

### Data Collection

The following information was extracted and tabled from each selected study: (1) authors' names, (2) sample, (3) task design, (4) acquisition system, (5) stimuli, and (6) results.

## Results

### Study Selection

The search engine found papers in 20 databases, as shown in Table 1; from those, eight had papers included in this research. Each paper was indexed in an average of 3.11 of the included databases (SD = 1.45). Science Citation Index Expanded from Web of Science was the database with more papers indexed in; the only paper that was not indexed by them was the one from Ganea et al. (2020).

**Table 1:** Databases found by the search engine.

**Database**	**Papers found**	**Papers included**
Scopus (Elsevier)^*^	1.478	4
OneFile (GALE)^*^	642	3
Science citation index expanded (Web of Science)^*^	537	8
Taylor & Francis Online—Journals	442	
Technology Research Database^*^	385	3
ERIC (U.S. Dept. of Education)	378	
Social sciences citation index (Web of Science)^*^	333	2
Advanced technologies & aerospace database	282	
Engineering research database^*^	280	3
SpringerLink	245	
Materials science & engineering database	235	
Arts & humanities citation index (Web of Science)	184	
PMC (PubMed Central)^*^	180	2
Directory of open access journals (DOAJ)^*^	175	3
JSTOR archival journals	157	
Sage journals (Sage publications)	148	
Cambridge journals (Cambridge University Press)	135	
Sociological abstracts	105	
Computer and information systems abstracts	103	
Emerald insight	64	

* *Databases that had papers included in this review*.

From the 6,488 papers found, 4,568 were duplicates and nine fulfilled the inclusion criteria, as shown in Figure 2.

### Studies' Demographics

**Figure 3:**
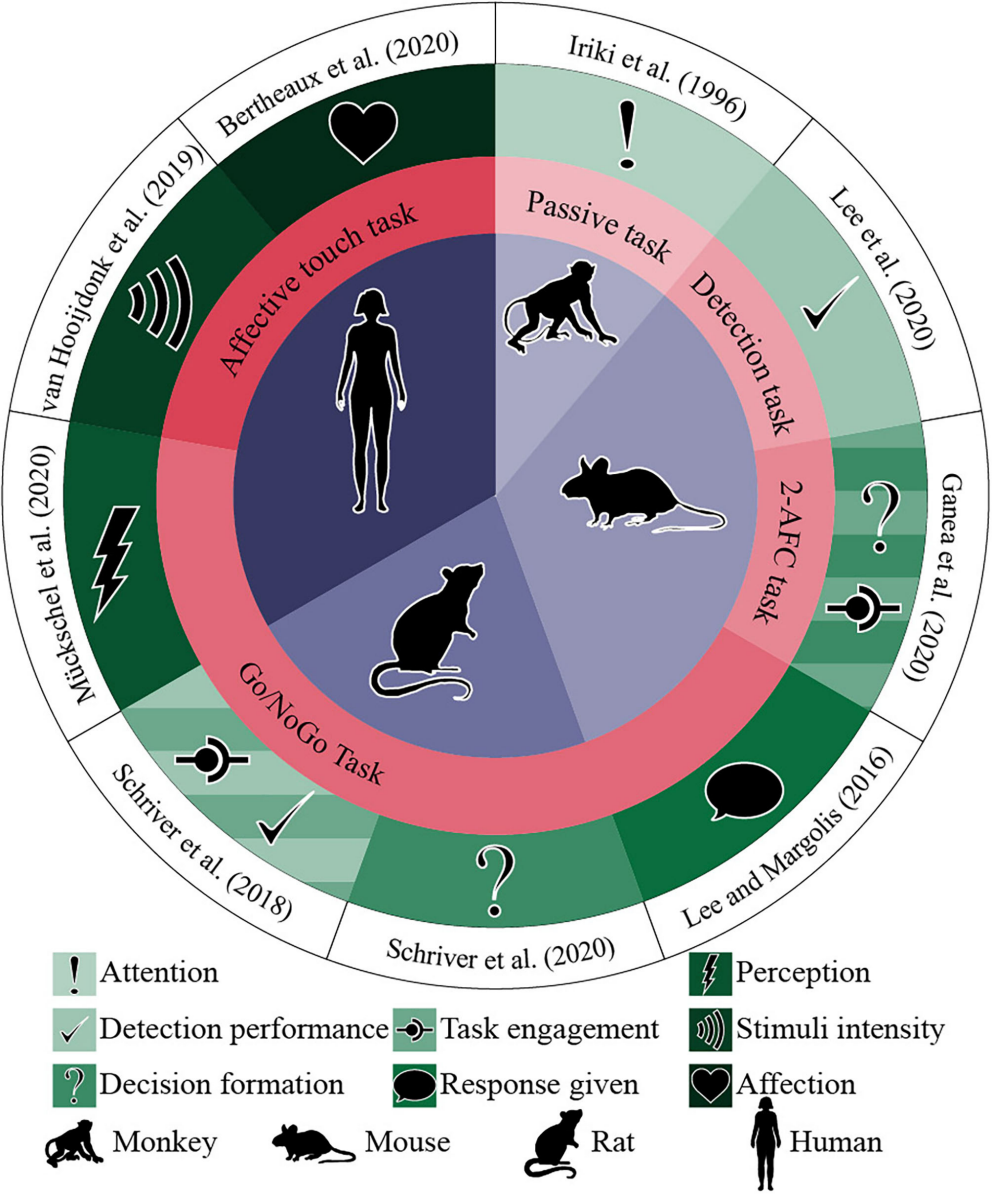
Studies, participants, task type, and main results. The circles from the center outwards represent (1) research participants (purple), (2) task type (coral), (3) positive correlation with pupil dilation (green), and (4) paper's authors and year (white).

As Table 2 and Figure 3 show, the nine studies were conducted between 1996 and 2020. Three used human subjects (two with adults and one with adolescents) (Van Hooijdonk et al., 2019; Bertheaux et al., 2020; Mückschel et al., 2020), one wild mice (Lee and Margolis, 2016), two with C57BL6J mice (Ganea et al., 2020; Lee et al., 2020), two albino rats (Schriver et al., 2018, 2020), and one juvenile male Japanese monkeys (*Macaca fuscata*) (Iriki et al., 1996). Four studies used go/no-go tasks (Lee and Margolis, 2016; Schriver et al., 2018, 2020; Mückschel et al., 2020), one a vibration detection task (Lee et al., 2020), one a two-alternative forced choice (2AFC) task (Ganea et al., 2020), one used a passive task (Iriki et al., 1996), and two discrimination of affective touch tasks (Van Hooijdonk et al., 2019; Bertheaux et al., 2020).

**Table 2:** Overview of included studies.

**References**	**Sample**	**Task design**	**Acquisition system**	**Stimuli**	**Results**
Bertheaux et al. (2020)	25 humans (12 males, 18–27 years old)	Discrimination of affective touch	ISCAN-ETL-100H	12 affective materials	Affective touch modulates pupil.
Ganea et al. (2020)	8 C57BL/6J mice (8 males, 3–4 months old^*^)	2AFC	Point gray Chameleon3 camera	Bending actuator	Pupil dilation reflects internal decision components.
Iriki et al. (1996)	2 *Macaca fuscata* (2 males, juvenile)	Passive stimulation	Motor only shot (MOS) camera	Probe to the fingers with a pressure transducer	Pupil dilation onset occurred before the somatosensory stimulus associated with the clue.
Lee and Margolis (2016)	6 wild mice (5 males, 63–79 days old)	Go/no-go task	Allied Vision Technologies Pike F-032 camera	120- and 1,200-grid sandpaper	Pupil dilation was related to response rather than stimuli.
Lee et al. (2020)	7 C57BL/6J mice (7 males, 4 weeks old)	Vibration detection task	DMK22BUC03	Piezo-driven mesh	Pupil dilation at baseline is related to detection performance.
Mückschel et al. (2020)	22 humans (8 males, 14.48 ± 0.21 years old)	Go/no-go task	RED 500	Miniature electromagnetic stimulator	Pupil diameter was larger for go than for no-go trials.
Schriver et al. (2018)	5 albino rats (5 females, 6–10 months old^*^)	Go/no-go task	In-house system (FL3-U3-13Y3M-C; FLIR)	Piezoelectric bending actuator	Pupil dilation at baseline is related to detection performance and reaction time.
Schriver et al. (2020)	8 albino rats (8 females, 6–10 months old^*^)	Go/no-go task	In-house system (FL3-U3-13Y3M-C; FLIR)	Piezoelectric bending actuator	Pupil dilation is influenced by stimulus encoding and decision formation.
Van Hooijdonk et al. (2019)	28 human adults (11 males, 19.14 ± 1.02 years old)	Discrimination of affective touch	EyeTribe tracker	Foundation brush	Touch-induced pupil size reflects stimulus intensity.

* *Information obtained via e-mail with the paper's contact author*.

### Pupil Diameter Acquisition Method

For acquiring the pupil diameter, seven different methods were used (Table 2). Iriki et al. (1996) did not report the equipment other than saying it was a motor-only shot (MOS) camera. The reported equipment were a 10-Hz in-house system (Schriver et al., 2018, 2020), 30-Hz EyeTribe tracker (Van Hooijdonk et al., 2019), Point Gray Chameleon3 camera (Ganea et al., 2020), DMK22BUC03 (Lee et al., 2020), 50-Hz Allied Vision Technologies Pike F-032 camera (Lee and Margolis, 2016), 60-Hz ISCAN-ETL-100H (Bertheaux et al., 2020), and 500-Hz RED 500 (Mückschel et al., 2020). All studies had controlled luminance and either excluded the trials where there were blinks or interpolated the data.

### Stimulus Sources and Their Technical Characteristics

Bertheaux et al. (2020) presented 12 different stimulus sources classified as follows: three unpleasant (different sandpaper roughness levels), seven neutral (three 100% polylactic material and four mixes of 50% polylactic material and 50% other materials), and two pleasant (velvet and synthetic fur). The participants should actively touch the materials with their dominant hand in three rounds of 15 s; the first one was not considered on data analysis to avoid the surprise element. Ganea et al. (2020) delivered different cosine waves to mice whiskers with a piezo bending actuator (Johnson Matthey, Royston, UK) amplified by a piezo controller (MDT693A; Thorlabs, NJ, USA). Schriver et al. (2018) and Schriver et al. (2020) also used a piezoelectric actuator (PL140, Physik Instrumente, Karlsruhe, Germany) driven by a high-voltage amplifier (OPA452; Texas Instruments, Dallas, TX). The 2018 paper used whisker deflections with durations of 25, 50, and 100 ms with respective velocities of ~1,200, 600, and 300°/s, while they did not report the duration/velocity of the deflections on the 2020 paper. Lee et al. (2020) delivered vibration to the left whisker pad via an aluminum mesh (2 × 2 cm) attached to a ceramic piezoelectric wafer (Morgan Matroc, Bedford, OH); they delivered a series of discrete Gaussian deflections: 15- vs. 10-ms pause with a frequency of 40 Hz and a total duration of 300 ms with five different amplitudes: 0, 10, 20, 40, and 80 μm. Iriki et al. (1996) used a probe to the fingers with a pressure transducer attached to record the stimulation's timing and strength. Lee and Margolis (2016) used 120- and 1,200-grid sandpaper. Mückschel et al. (2020) delivered 150 and 40 Hz sinewave stimulus via a miniature electromagnetic stimulator (Dancer Design; for detailed information, see http://www.dancerdesign.co.uk). Lastly, Van Hooijdonk et al. (2019) used a foundation brush (goat hair; conducted pressure ~11.5 Pa). In personal contact with the author, they clarified that the experimenter stroked the participant's forearm with a foundation brush. The timing was controlled by marking a distance on the arm and stroking it in the required time to achieve the velocity of that condition (e.g., 6-cm distance requires stroking of 2 s) (Table 2).

### Results of the Selected Studies

All studies indicated a positive correlation between stimuli perception and pupil dilation (Table 2). As shown in Figure 3, while Bertheaux et al. (2020) found that neutral materials lead to a lower pupil dilation than materials with high emotional intensity, independently of their valence (being pleasant or unpleasant), Van Hooijdonk et al. (2019) associated the difference in the pupil dilation only with the stimulus intensity, not subjective pleasantness. The four go/no-go tasks (Lee and Margolis, 2016; Schriver et al., 2018, 2020; Mückschel et al., 2020) and the 2AFC task (Ganea et al., 2020) found a significantly broader pupil dilation for go trials than no-go ones. Lee and Margolis (2016) and Ganea et al. (2020) also found that not only the correct behavior had a bigger pupil but also the false alarms, associating the pupil dilation with the type of behavioral response rather than the type of stimulus presented. Ganea et al. (2020) and Schriver et al. (2018) showed that the pre-stimulus pupillary size reflected task engagement. Iriki et al. (1996) found that primary sensory area (SI) neurons responded to a light touch of the glabrous skin, two neurons to manipulation of finger joints; during the period, no pupil dilation was induced (monkey was not attentive). When attentional dilation was induced during the task, another 10 neurons responded to the glabrous skin's stimulation. Lee et al. (2020) calculated cross-correlation between pupil diameter and detection performance. It was computed from the average of pupil dilation and behavior in a five-trial sliding window. They found a positive correlation between detection performance and pupil dilation, with pupil diameter lagging behind performance by a median of 9.2 trials. They also found that the pupil dilation's amplitude was positively influenced by the licking, response time, and detection rate.

## Discussion

### Pupil Diameter Acquisition Method

The selected studies' sampling rate range was between 10 and 500 Hz (Table 3), even with most of them having a small sampling rate—modern eye-trackers can achieve 2,000 Hz—they have proved that pupil dynamics can be seen as either an impoverished measure of brain function or a rich measure of cognition as Eckstein et al. (2017) has previously defended. While the 30-Hz systems (Van Hooijdonk et al., 2019; Ganea et al., 2020; Lee et al., 2020) are the slowest commercially available, there was an in-house-developed system that was only 10 Hz (Schriver et al., 2018, 2020). Typically used are 50 and 60 Hz (Lee and Margolis, 2016; Bertheaux et al., 2020) since these are the most common frequencies in camera technology for a long time. The 500 Hz (Mückschel et al., 2020) started to be used in 2000 and, from the studies found in this research, is the best sampling rate. The ideal sampling rate, especially when analyzing eye movements, is > 250 Hz; no mathematical method defines a cut-off value, but it instead has been established through consensus (Holmqvist et al., 2011). Since equipment with low sampling rates is cheaper, it is broadly used.

**Table 3:** Number of times each dataset would have to be larger than a dataset with a 250-Hz sampling rate.

**References**	**SR (Hz)**	**Ideal**	***N***	***t*/*s***	***s***	**Total**
Bertheaux et al. (2020)	60	17.36	25	12	1	300
Ganea et al. (2020)	30	69.45	8	336.2	11.5	30,930
Iriki et al. (1996)	14.29	204	2	350?	N/A	N/A
Lee and Margolis (2016)	50	25	6	~106	~4	2,766
Lee et al. (2020)	30	69.45	7	300–400	5^*^	~13,400^*^
Mückschel et al. (2020)	500	0	22	208	4	18,304
Schriver et al. (2018)	10	625	5	~344	~22	38,249
Schriver et al. (2020)	10	625	8	~300^*^	~24	~57,000^*^
Van Hooijdonk et al. (2019)	30	69.45	28	2	27	1,512

*Column three (ideal) shows how many times the data set should be increased to get to the ideal amount necessary for reliable results based on Holmqvist et al. (2011). n, number of participants; t, trials; s, sessions. Total is the overall total of trials. Question marks (?) represent inferred data from the paper (not presented clearly by the authors)*.

* *Information obtained via e-mail with the paper's contact author*.

Holmqvist et al. (2011) stated that a low sampling rate could be compensated for by enlarging the number of data acquired in a quadratic manner. This way, data acquired by 10 Hz (SRa) would need to have 625 times more data to have the same quality as a study at 250 Hz (the minimum ideal sampling rate—SRi). As shown in the following equation:

$$\begin{array}{l} {x=\;\left(\frac{\text{SRi}}{\text{SRa}}\right)}^{2} \end{array}$$

Table 3 shows how many times each dataset would have to be larger than a dataset with a 250-Hz recording and the total number of trials for each study presented here. Four of the works with a small sampling rate had a total number of trials bigger than 10,000 (Schriver et al., 2018, 2020; Ganea et al., 2020; Lee et al., 2020), which should compensate for the low recording sampling rate. Meanwhile, three other studies had fewer trials (Lee and Margolis, 2016; Van Hooijdonk et al., 2019; Bertheaux et al., 2020). Bertheaux et al. (2020) had only 300 trials total, and for Iriki et al. (1996), data were not available. The three studies with fewer trials are still analyzed here, but their results should be taken carefully and validated through coherence with the other studies.

### Stimulus

The automated systems, as in the four go/no-go, the vibration detection task, and the 2AFC tasks (Lee and Margolis, 2016; Schriver et al., 2018, 2020; Ganea et al., 2020; Lee et al., 2020; Mückschel et al., 2020) for stimuli deliverance are the most reliable since they depend neither on the participants' interaction with the object (Bertheaux et al., 2020) nor the researcher presenting the stimulus (Iriki et al., 1996; Van Hooijdonk et al., 2019). Participant's interaction may be a confounder since the researchers cannot dissociate the tactile perception from movement results. The studies on which the researcher was responsible for presenting the stimuli could lead to human flaws that Iriki et al. (1996) accounted for when they added a pressure transducer to the stimulation's strength and timing, but not reported by Van Hooijdonk et al. (2019). The range of stimuli in the studies and the lack of automated systems reflect the reality of the field and the fact that there is less research relating tactile perception to pupil dilation than visual or auditory (Aston-Jones and Cohen, 2005; Einhauser et al., 2010; Piquado et al., 2010; Laeng and Endestad, 2012; Kang and Wheatley, 2015; Wetzel et al., 2016; Eckstein et al., 2017)—tactile stimuli are harder to deliver and to monitor.

### Aims and Results of the Selected Studies

Although all papers linked pupil dilation and tactile perception, they had different aims, and from those aims came different interpretations of the results, showing the multifaceted character of pupil-related research.

Bertheaux et al. (2020) and Van Hooijdonk et al. (2019) tried to find the relation between pupil dilation and pleasantness of the stimulus. Both found that dilation is related to perception, but they had different approaches and results interpretation. Bertheaux et al. (2020) found that when exploring different materials—not considering the first pupil dilation related to the surprise of the touch—the pupil was modulated by the affect. As in auditory and visual studies, both pleasant and unpleasant stimuli led to larger pupil dilation than neutral ones (Partala and Surakka, 2003; Bradley et al., 2008). Van Hooijdonk et al. (2019) used a foundation brush to deliver stimulus for 15 s in different velocities, considering that the mean velocity (3 cm s^−1^) was considered a pleasant one compared to two other neutral velocities (0.3 and 30 cm s^−1^). They analyzed data from the moment the brush touched the skin and found that pupil dilation was influenced by the stroke's velocity rather than its pleasantness. Opposite to Bertheaux et al. (2020), they state that any sympathetic response to affective touch is probably too small, leading to it being overshadowed by the sympathetic response due to increasing the amount of A-beta tactile input (touch itself). From these two studies, we can hypothesize that the relationship with the stimulus' pleasantness can be divided into two moments: a first moment of arousal, when pupil dilation is related to the stimulus' strength, and a second moment of exploration, when it is emotionally modulated—also asserted by Bradley et al. (2008). Studies from the visual and auditory fields relate pupil dilation with affective stimulus (Partala and Surakka, 2003; Bradley et al., 2008), but those two tactile studies are still incomplete. Further studies associating pleasantness of a stimulus and pupil dilation should be developed. Unlike the two here reported, they should automate the yielded stimuli, recording with a higher sampling rate and a larger amount of trials, and integrate both early and later pupillary reactions to verify the hypothesis formulated here.

Iriki et al. (1996) used the pupil as an objective indicator of attention. Although they did not analyze the effect of the tactile stimuli on the pupil, they found that when the pupil dilation indicated attention, more SI neurons responded to the stimulation of the glabrous skin. They defend that the somatosensory cortex is an active processor that controls the perceptual processes depending on the current behavioral context. In a similar interpretation of data, Schriver et al. (2018) found that the pupil baseline positively correlated to the behavior outcome; they determined that the sensitivity peak was achieved with intermediate pupil baselines. These results are consistent with a visual task that found that tonic arousal influenced early target selection signals and decision formation consistency; in contrast, phasic arousal affected behavior through its relation to attentional engagement and decision formation consistency (Van Kempen et al., 2019). A speech perception study (Ayasse and Wingfield, 2019) stated that the baseline pupil reflects a heightened arousal level in poorer-hearing participants that dissipated over trials. They suggest that the baseline pupillary response may also be connected with the effort necessary or expected related to the task and not only a single construct.

Even with different aims and results, the four go/no-go and the 2AFC tasks considered the importance of the norepinephrine (NE) and locus coeruleus (LC) on the pupil dilation process. Lee and Margolis (2016) and Ganea et al. (2020) aimed to determine the pupil dynamics association with task performance and found out that pupil dilation was present both on hit and false alarm. They correlated the increase in dilation with the sensory processing and preparation for action, and they were able to predict the choice with 80% precision. Ganea et al. (2020) also found that pre-stimulus pupillary size reflected mice engagement on the task; they were able to predict if mice would report perceiving the stimulus for both hit and false alarm, which is congruent with Aminihajibashi et al.'s (2020) findings in a visual task, with which they concluded that the pupil appears to be affected by arousal, rather than attention itself, since its reaction to a cue was not a predictor of better performance. Interestingly, Schriver et al. (2018) found biexponential curve shapes for pupil dilation similar to the one elicited by phasic LC activation by Liu et al. (2017) and Joshi et al. (2016) for the hit, miss, and correct reject trials, but not for false alarm ones, which showed a plateau pattern that still needs investigation for understanding its cause.

A not-yet-published work by Yang et al. (2020) simultaneously measured the LC and cortical activity and pupil diameter during a go/no-go single whisker detection task. They found that the LC spiking activity and pupil dilation were related during all the trials. As hypothesized by the studies presented here (Lee and Margolis, 2016; Schriver et al., 2018, 2020; Ganea et al., 2020), they also found a spike on LC aligned to “go” responses (hits and false alarm trials). Another finding was spiking related to the tone that indicated the beginning of a new trial; this activity was lower for hits than misses, with the pupil diameter being larger for the latter on the baseline period. These findings are similar to Iriki et al. (1996) but differ from Schriver et al. (2018) that found that intermediated pupil baselines corresponded to perceptual sensitivity peak in a quadratic/non-linear manner. They also found that LC spikes preceded SI depolarizations and pupil dilations. LC's spiking correlated with both Vm and pupil diameter changes, but on vastly different timescales, they also showed that the time derivative of pupil diameter, but not the absolute pupil size, is a good predictor of SI Vm fluctuations (Yang et al., 2020).

Instead of considering the “go” trials, Mückschel et al. (2020) emphasized the inhibition process of the “no-go” trial. They found that for adolescents—who typically express more difficulty inhibiting SI stimuli (Bodmer et al., 2018)—there was a positive correlation between pupil size and SI inhibition. While Lee and Margolis (2016) associated their findings with the engagement of arousal systems as NE LC neurons linked to onset cues, Ganea et al. (2020) associated their findings with LC-NE adaptive gain theory, and Mückschel et al. (2020) affirm that the NE system is not well-inhibited by lower-level processing of SI stimulation in adolescents.

### Equivocated Results Interpretation

Scientists should not jump to conclusions based on their judgment. The Universal Declaration on Bioethics and Human Rights states that “no individual or group should be discriminated against or stigmatized on any grounds, in violation of human dignity, human rights and fundamental freedoms” (Unesco, 2005). Nonetheless, Bertheaux et al. (2020) brought two biased interpretations. Two of their participants considered synthetic animal fur unpleasant, and they interpreted that it was because the participants grew up in countries where animals are dangerous. They inferred this from their nationality with no recorded inquisition about why they considered the fur unpleasant. They did not consider factors as familiarity, fluency, typicality, and complexity that Carbon and Jakesch (2012) listed as relevant for emotional and cognitive haptic processing. They also found differences in pupil dilation between men and women, which they attributed to the fact that “sandpaper is mainly used by men in construction; manufacturing and its surface appearance may seem more familiar to them” (p. 11). There is evidence that men and women have different pupil dilation patterns, not necessarily associated with the kind of tactile stimulation received; those patterns can predict gender with 90% accuracy (Costa-Abreu et al., 2015)—this study was not included in this revision since it is only an abstract.

### Future Perspectives

More studies correlating tactile stimulation and pupillometry should be done, mostly avoiding the confounders as cue signs, not automated stimulation, low recording sampling rate, and concurrent stimulus. We suggest using threshold tasks, already explored in vision (Pins and Ffytche, 2003; Ress and Heeger, 2003; Wyart and Tallon-Baudry, 2008; Herman et al., 2019) and audition (Colder and Tanenbaum, 1999; Christison-Lagay et al., 2018).

Due to the importance of developing self and improving social relations (Bremner and Spence, 2017), the study of affective touch still has a long way to go. One of the major problems found in the study of Van Hooijdonk et al. (2019) was that the stimuli were delivered manually with a foundation brush. The same kind of stimuli has already been delivered using robotic systems to ensure its precision (Loken et al., 2011; Croy et al., 2014; Sailer et al., 2016; Hielscher and Mahar, 2017) and should also be applied in the pupillometric research.

The go/no-go studies seem to be a robust and reliable tool for the study of tactile perception. Nonetheless, most of the studies presented here used low sampling rate recording systems (Lee and Margolis, 2016; Schriver et al., 2018, 2020). The acquisition method should be more similar to Mückschel et al. (2020) (sampling rate ≥250 Hz), but with a more in-depth analysis of the variances, especially at the baseline period as in Schriver et al. (2018), and taking into account the multi-factor process of perception and report (e.g., stimulus encoding and decision formation) as in Schriver et al. (2020).

Based on the studies found here and the ones from the visual and auditory field, pupillometry can be an indirect indicator of cognitive processes. Since pupil dynamics seem to represent a potentially powerful, relatively inexpensive—but still reliable and robust—tool for gauging perceptual and cognitive processing in the absence of an overt perceptual report, it leverages these metrics in the development of no-report paradigms in future studies across sensory modalities. Such studies would allow the research of consciousness in a more “pure” way since it will not have confounder facts as the activity of memorizing the stimulus and getting ready to answer a question (Colder and Tanenbaum, 1999; Pins and Ffytche, 2003; Ress and Heeger, 2003; Luna et al., 2008; Einhauser et al., 2010; Piquado et al., 2010; Laeng and Endestad, 2012; Tatler et al., 2014; Kang and Wheatley, 2015; Eckstein et al., 2017; Christison-Lagay et al., 2018; Aminihajibashi et al., 2020; Ganea et al., 2020).

For confirmation of the information found here, we suggest reproducing the low sampling rate studies with a higher one (Lee and Margolis, 2016; Schriver et al., 2018, 2020; Van Hooijdonk et al., 2019; Bertheaux et al., 2020; Ganea et al., 2020; Lee et al., 2020). Also, since Lee and Margolis (2016) and Ganea et al. (2020) found that the pupil can reliably predict behavior, more studies should be done with no-report tasks.

We also propose more research in the different steps of haptic processing, not only on lower levels but also on mid and high-level processing, as discussed by Carbon and Jakesch (2012).

## Conclusion

The assessed literature confirms that pupil dilation is a reliable indirect measure of brain states and can evaluate LC/NE action, stimulus inhibition, arousal, cognitive processes, and affection. During a tactile task, pupil dilation reflects both tonic and phasic changes on the LC that can be associated with the perception itself, independently of the stimuli's nature (Colder and Tanenbaum, 1999; Pins and Ffytche, 2003; Ress and Heeger, 2003; Wyart and Tallon-Baudry, 2008; Christison-Lagay et al., 2018; Herman et al., 2019) More studies are required to confirm these investigations' results (as some have been done with relatively low sampling rates).

## Data Availability Statement

The original contributions presented in the study are included in the article, further inquiries can be directed to the corresponding author/s.

## Author Contributions

MG was responsible for the conception of the review, literature research, and manuscript writing. GS was responsible for literature research and paper review. PN supervised the selection of the studies and contributed to the revision of the manuscript. All authors revised, read, and approved the submitted version.

## Conflict of Interest

The authors declare that the research was conducted in the absence of any commercial or financial relationships that could be construed as a potential conflict of interest.
